# Partial facial paralysis induced by sialolithiasis of the parotid gland: a case report

**DOI:** 10.1186/s12883-024-03602-6

**Published:** 2024-03-22

**Authors:** Abhinav Suri, Stephen Avila, Christina Tan, Huda Alalami, Jennifer Harris

**Affiliations:** 1 David Geffen School of Medicine at UCLA, 10833 Le Conte Ave, Los Angeles, CA 90095 USA; 2 Cedars Sinai, Department of Neurology, 8700 Beverly Blvd., Los Angeles, CA 90048 USA

**Keywords:** Facial paralysis, Parotid sialolithiasis, Parotitis, CN VII lesion

## Abstract

**Background:**

Facial paralysis due to parotid sialolithiasis-induced parotitis is a unusual clinical phenomenon that has not been reported in prior literature. This scenario can present a diagnostic challenge due to its rarity and complex symptomatology, particularly if a patient has other potential contributing factors such as facial trauma or bilateral forehead botox injections as in this patient. This case report elucidates such a complex presentation, aiming to increase awareness and promote timely recognition among clinicians.

**Case presentation:**

A 56-year-old male, with a medical history significant for hyperlipidemia, recurrent parotitis secondary to parotid sialolithiasis, and recent bilateral forehead cosmetic Botox injections presented to the emergency department with right lower facial drooping. This onset was about an hour after waking up and was of 4 h duration. The patient also had a history of a recent ground level fall four days prior that resulted in facial trauma to his right eyebrow without any evident neurological deficits in the region of the injury. A thorough neurological exam revealed sensory and motor deficits across the entirety of the right face, indicating a potential lesion affecting the buccal and marginal mandibular branches of the facial nerve (CN VII). Several differential diagnoses were considered for the lower motor neuron lesion, including soft tissue trauma or swelling from the recent fall, compression due to the known parotid stone, stroke, and complex migraines. An MRI of the brain was conducted to rule out a stroke, with no significant findings. A subsequent CT scan of the neck revealed an obstructed and dilated right Stensen's duct with a noticeably larger and anteriorly displaced sialolith and evidence of parotid gland inflammation. A final diagnosis of facial palsy due to parotitis secondary to sialolithiasis was made. The patient was discharged and later scheduled for a procedure to remove the sialolith which resolved his facial paralysis.

**Conclusions:**

This case emphasizes the need for a comprehensive approach to the differential diagnosis in presentations of facial palsy. It underscores the potential involvement of parotid sialolithiasis, particularly in patients with a history of recurrent parotitis or facial trauma. Prompt recognition of such uncommon presentations can prevent undue interventions, aid in timely appropriate management, and significantly contribute to the patient's recovery and prevention of long-term complications.

## Background

Facial paralysis, characterized by sudden, unilateral weakness or loss of facial muscle function, presents a diagnostic challenge for clinicians due to its myriad potential causes. The differential diagnoses typically include conditions such as Bell's palsy, stroke, Lyme disease, Ramsay Hunt syndrome, and idiopathic peripheral facial nerve palsy. However, rare causes like parotid sialolithiasis-induced parotitis leading to facial nerve compression and resulting in facial paralysis are not commonly explored.

Parotid sialolithiasis refers to the formation of salivary stones in the parotid gland, often leading to recurrent episodes of parotitis or inflammation of the parotid gland. Although parotid sialolithiasis is a well-recognized condition, it is relatively rare, accounting for only 15% of all salivary gland stones [1, 2]. The main symptoms usually revolve around pain, swelling, and recurrent infections, but facial palsy has been not previously been reported as a manifestation.

The aim of this case report is to fill this knowledge gap by presenting a unique case of facial palsy resulting from parotid sialolithiasis-induced parotitis. This report will provide a comprehensive examination of the patient's history, presentation, diagnosis, and treatment. By doing so, it aims to enhance the understanding of this unusual cause of facial paralysis, raising awareness among clinicians and aiding in timely diagnosis and appropriate treatment of similar future cases.

## Case presentation

A 56-year-old male with a past medical history of hyperlipidemia, recurrent parotitis secondary to parotid sialolithiasis, and recent bilateral forehead cosmetic botox injection presented to the emergency department with a 4-h history of right lower facial drooping approximately 1 h after waking up (he reported sleeping on the opposite side). The patient had a ground level fall 4 days prior, caused by slipping on the ground, resulting in facial trauma that presented with laceration of the right lateral eyebrow but no loss of consciousness or any neurological symptoms (sensation, motor function intact in region). The patient did not report any recent infections or infectious symptoms prior to presentation and work up for causes of recurrent parotitis (ruled out malignancy, auto-immune conditions, tuberculosis, HIV, diabetes, primary hyperparathyroidism) that determined it was idiopathic.

Neurological exam revealed sensory deficits across the entirety of the right face and motor deficits especially evident in the upper and lower right lip when smiling (House Brackmann Score III). The presence of upper facial involvement was unclear due to forehead paralysis possibly secondary to Botox. The patient had no auditory or taste abnormalities. The patient had no other motor deficits (5/5 strength in all extremities) and no signs of cerebellar dysfunction, such as dysmetria or ataxia. Deep tendon reflexes were symmetrical and normal (2 +).

Given these exam findings, it was most likely this individual has a lower motor neuron lesion of CN VII specifically as opposed to a stroke (no evidence of cortical signs of stroke, and other areas of focal weakness). Initial differential localized the lesion to the buccal and possibly marginal mandibular branches of CN VII given his inability to smile. It was unclear as to whether or not he had upper facial involvement; however, the lack of involvement of zygomatic branches (sparing of orbicularis oculi muscle given ability to keep eyes closed to resistance.

In terms of differentials to consider as etiologies for CN VII lesions: this patient does have a recent trauma to his face due to a ground level fall which could cause soft tissue swelling and compression of the aforementioned CN VII. Additionally, the patient has a known parotid stone which could also contribute to soft tissue swelling of the parotid gland and surrounding neurovascular structures. Lower on the differential is a stroke given lower facial droop that lateralizes (though very unlikely at this point in the presentation, but nonetheless critical to rule out) or complex migraine with sensory changes and weakness (though the patient did not have a headache on presentation or history of migraine). Other etiologies considered were Ramsay Hunt syndrome (though no history of herpes simplex virus in this patient and additionally no auditory abnormalities) and Lyme disease (though no recent travel, illnesses, tick bites, or rashes).

MRI Brain was obtained to rule out stroke as an explanation for his current symptoms. The resulting read was normal. The neurology team also ordered a CT neck without contrast to evaluate for parotitis as an etiology for his current symptoms of facial paralysis given suspicion for a compressive CN VII neuropathy secondary to parotitis.

**Fig. 1:**
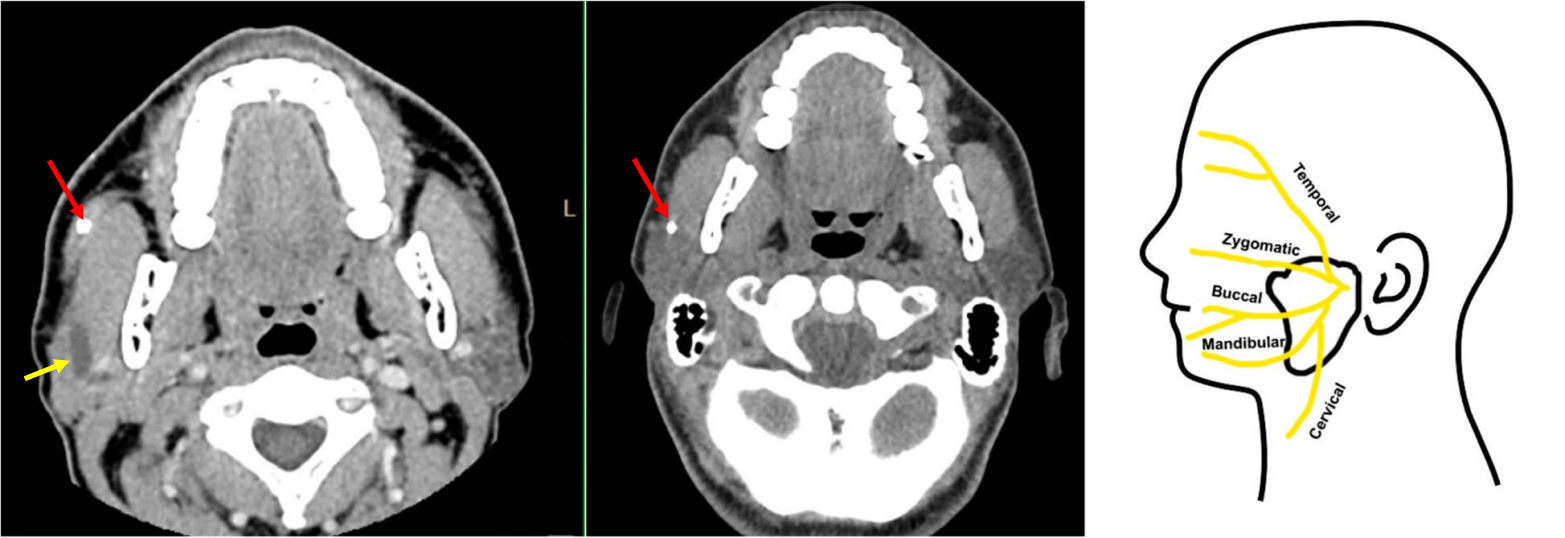
First two panels: CT Head, third panel: anatomical representation of branches. On the left showing CT neck from current admission and in the middle showing CT neck from 6 months ago. Red arrows point to stone location. Yellow arrow points to dilated Stensen’s duct

CT neck showed an obstructed and dilated right Stensen’s duct extending into the deep right parotid gland (Fig. 1). Obstructing the duct was a sialolith that had grown in size (from 3 to 6 mm) and had been more anteriorly displaced compared to prior scans. Furthermore, there was evidence of soft tissue swelling of the right parotid gland in comparison to the left parotid. Given that the stone was evident on CT and patient had already arranged for ENT follow up to remove the stone, MR was deferred.

Subsequent evaluation of the patient four hours later had shown that the degree of right upper lip paralysis had improved significantly compared to prior presentation. Given the presence of a larger, more anteriorly displaced stone (possibly dislodged after his recent ground level fall) causing soft tissue inflammation of the right parotid and ductal structures in the gland, it was deemed likely that the patient’s symptoms were most likely due to parotitis secondary to sialolith as opposed to stroke or bell’s palsy. The patient was then discharged to home. At a follow up visit with an ENT physician, clinical exam confirmed parotitis and the patient was scheduled for a procedure to remove his sialolith in the following weeks. Subsequent removal of the stone (endoscopic removal) resolved this patient’s facial palsy without recurrence in the year after his initial presentation.

## Discussion and conclusions

This patient had CN VII palsy likely secondary to sialolithiasis induced parotitis/CN VII nerve compression. In a survey of current literature, many cases have been reported of parotitis causing facial nerve palsy; however, existing literature/case reports focus on etiologies ranging from abscess, tumor, post-surgical complications, rare cases of amyloidosis, or sarcoidosis [3–6]. In this case, the pathophysiology was likely secondary to direct mechanical compression of branches of the facial nerve by the parotid stone or secondary inflammation causing nerve entrapment. Given that the patient also had a prior fall resulting in a facial laceration, post-traumatic neuropraxia secondary to facial edema may have also contributed to his symptoms.

The incidence of parotid sialolithiasis is relatively low, estimated as low as 1 in 30,000 individuals and accounting for approximately 15% of all salivary gland stones [1, 2]. Factors contributing to the formation of parotid stones include dehydration, salivary stasis, and increased salivary calcium content [7]. In some cases, a history of recurrent sialadenitis or salivary gland infections may predispose an individual to develop sialolithiasis [7]. The majority of patients with parotid stones present with symptoms such as pain, swelling, and recurrent infections; however, facial palsy is a rare manifestation, and, to our knowledge, has not been reported in the literature before.

Definitive treatment of parotid sialolithiasis involves either extracorporeal shock-wave lithotripsy, sialoendoscopy, laser lithotripsy, or video assisted surgical removal of parotid calculi [8]. Conservative treatment includes NSAIDs and sialogogues [9, 10] For parotid stones that are mobile and measuring less than 7 mm (as was the case for this patient), endoscopic management is recommended for definitive treatment [10–12]. If unsuccessful, then extracorporeal shock-wave lithotripsy is used to break up the stone and facilitate the removal of the smaller stones via endoscopy.

Parotid gland surgery is considered to be the last line of treatment if all else fails, but this surgery unfortunately carries the risk of facial nerve palsy/damage [11]. Potential complications of parotid stone surgery include facial nerve injury, salivary duct injury, infection, and recurrence. Early diagnosis and intervention can lead to a favorable outcome, with minimal complications [13].

This case highlights the importance of considering parotid stone-induced facial palsy in the differential diagnosis of patients presenting with facial paralysis and concomitant parotid gland symptoms. Prompt recognition and appropriate management can lead to complete recovery and prevent long-term complications.

## Data Availability

Data sharing is not applicable to this article as no datasets were generated or analysed during the current study.

## Abbreviations

MR/MRI: Magnetic resonance imaging
CT: Computed Tomography (scan)
CN: Cranial Nerve
ENT: Ear-Nose-Throat
